# Peritumoral edema in meningiomas: a review of influencing factors, mechanisms, and management

**DOI:** 10.3389/fonc.2026.1740332

**Published:** 2026-02-02

**Authors:** Gaocai Zhang, Erman Wu, Yandong Li, Yongtao Zhang, Minghao Lian, Dangmurenjiafu Geng, Guohua Zhu

**Affiliations:** Neurosurgery Center of the First Affiliated Hospital of Xinjiang Medical University, Urumqi, Xinjiang, China

**Keywords:** aquaporins, HIF-1, mast cells, meningiomas, MMPs, PTBE, tenascin C, VEGF

## Abstract

Peritumoral brain edema is an accompanying symptom of meningiomas, and its severity impacts patient symptoms and prognosis. Meningioma-related peritumoral brain edema can result in severe symptoms such as neurological disturbance and brain herniation. Traditionally, the main treatment options for peritumoral brain edema in the perioperative period have been osmotherapy and corticosteroids, but the side effects and limited effectiveness cannot be ignored. This review summarizes the known influencing factors and mechanisms that contribute to meningioma-related brain edema, discusses the limitations of existing edema treatments, and outlines future edema treatments. More research on meningioma-related peritumoral brain edema is needed to improve patient outcomes and enhance treatment efficacy.

## 1 Introduction

Meningiomas represent the most frequently diagnosed primary intracranial tumors, accounting for approximately 14%–20% of all tumors arising within the cranial cavity (1). Currently, about 40,000 people are diagnosed with meningiomas worldwide every year, with a prevalence rate of around 97.5 per 100,000 people, and females are more frequent than males (2). According to the World Health Organization (WHO) classification of tumors in the central nervous system (CNS), meningiomas are categorized as WHO grade I (benign, making up about 80-85%), WHO grade II (atypical, around 10-15%), and WHO grade III (malignant, approximately 1-3%) (3). Most meningiomas are benign and grow indolently, but they can occur anywhere with arachnoid elements. Depending on growth location and tumor size, they can display headaches, memory loss, visual impairments, and even seizures (4).

Peritumoral brain edema (PTBE) is closely associated with meningiomas, with more than 60% of meningiomas exhibiting PTBE (5, 6). The accumulation of brain tissue fluid due to PTBE increases intracranial pressure, worsens neurological damage, causes severe complications (brain tissue displacement and herniation), complicates the treatment, and significantly affects the patient’s prognosis (4, 7, 8). The pathogenesis of meningioma-associated PTBE remains unclear. Multiple studies have demonstrated that PTBE is associated with clinical characteristics such as tumor location and size (9, 10) and histological subtypes (11–13). Molecular markers such as vascular endothelial growth factor (14–16), aquaporins (17–20), metalloproteinases (21, 22), and interleukin-6 (23–26) also show associations with PTBE. PTBE results from the interaction of several factors, but its mechanism remains unclear.

The main treatment approaches for peritumoral brain edema in the perioperative period have been osmotic therapy and steroids, but the side effects and limited effectiveness cannot be ignored (27–29). Developing new drugs and strategies for treating PTBE is an urgent priority. This review summarizes the currently known influencing factors and mechanisms leading to meningioma-associated brain edema, discusses the limitations of existing treatments for PTBE, and provides an outlook on the future prospects for treating PTBE.

## 2 Pathophysiological mechanisms of peritumoral edema

**Figure 1:**
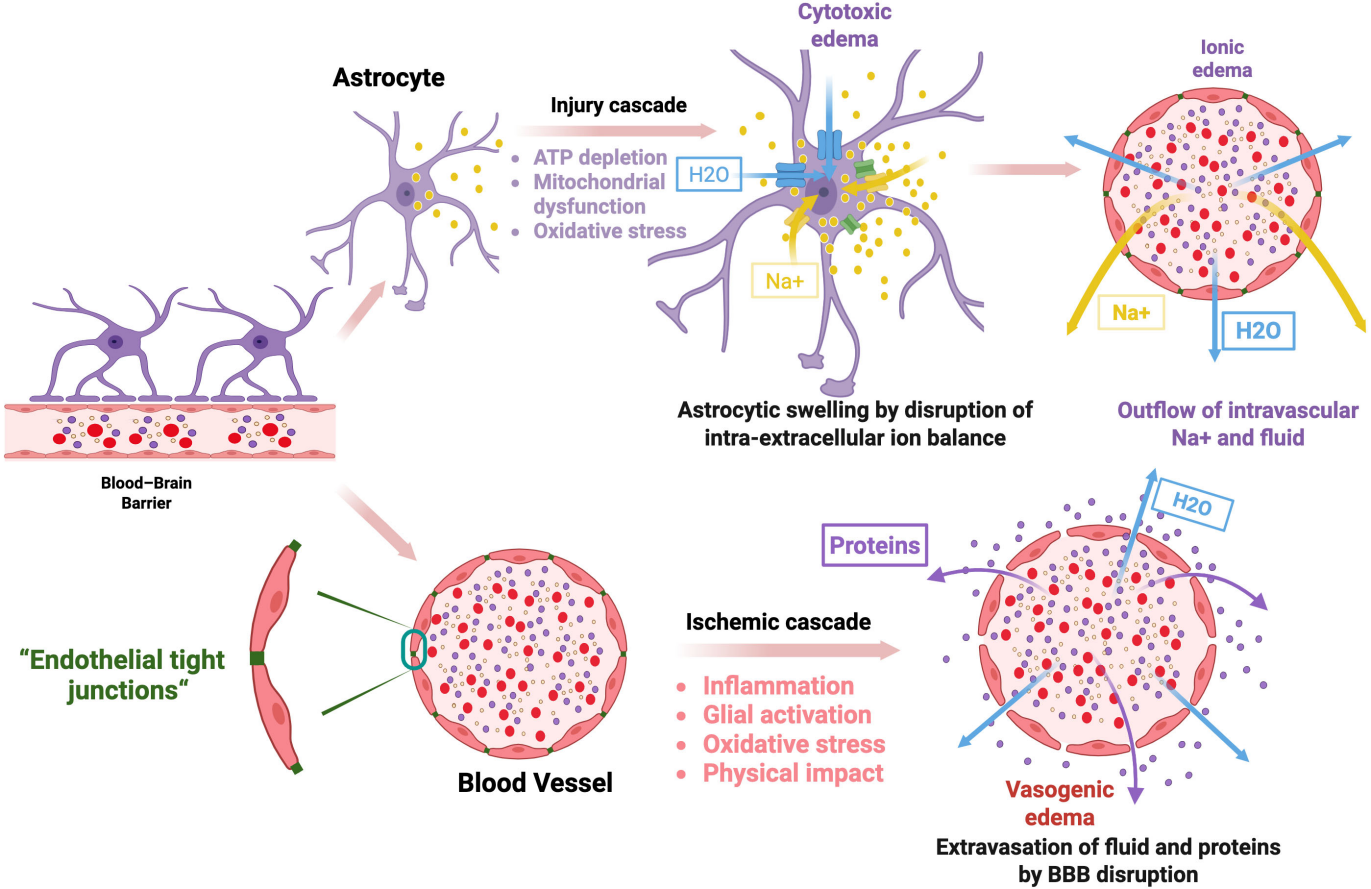
The pathophysiological mechanisms of vasogenic and cytotoxic edema. (1) Vasogenic Edema: Brain injury triggers ischemic cascades (excitotoxicity and oxidative stress) that disrupt endothelial tight junctions. Activated astrocytes and microglia release permeability factors and inflammatory mediators, increasing BBB permeability. As a result, fluid and proteins leak from vessels and build up in the brain’s extracellular space. (2) Cytotoxic Edema: Cerebral ischemia or hypoxia impairs energy metabolism, causing ATP depletion, mitochondrial dysfunction, and oxidative stress. Failure of ATP-dependent ion pumps leads to intracellular Na^+^ and Ca²^+^ accumulation, driving water influx and cell swelling. Ca²^+^ overload activates inflammatory and cytotoxic pathways, damages capillary membranes, and, together with reduced extracellular Na^+^, promotes sodium and water movement into brain tissue, resulting in ionic edema. Blue arrows: water flow, yellow arrows: Na+ flow, purple spheres: proteins, yellow spheres: Na+, blue columns: water channels, green columns: ion transporters, yellow columns: ion channels.

Since 1984, the pathophysiological mechanisms of brain edema have been primarily categorized into two types (**Figure 1**): vasogenic edema and cytotoxic edema (30, 31). The key distinction between these two lies in whether the blood-brain barrier (BBB) is compromised. The BBB is a selective membrane between brain tissue and blood, made of endothelial cells, astrocyte end-feet, and pericytes (32). The BBB precisely controls substance exchange in the CNS and protects the homeostasis of its internal environment (33).

### 2.1 Mechanisms of vascular edema

When brain tissue undergoes injury and pathological changes, the BBB undergoes reversible or irreversible destruction, leading to increased permeability that causes vasogenic edema. Following brain tissue injury, ischemia-reperfusion induces excitotoxicity and oxidative stress via mitochondrial dysfunction, a process termed the ischemia cascade (31, 32, 34). The above process can directly damage the constituent cells of the BBB, leading to irreversible injury. Additionally, the ischemic cascade induces leukocyte migration and activates glia, such as astrocytes and microglia, to secrete vascular permeability factors, cytokines, and chemokines, further increasing the endothelial cell and tight-junction permeability in the BBB (4, 31). Disruption and increased permeability of the BBB cause fluid and protein leakage into brain tissue, raising intracranial pressure (31).

### 2.2 Mechanisms of cytotoxic edema

Cytotoxic edema is characterized by abnormal accumulation of fluid in the brain cells, leading to swelling. This can occur in cerebral ischemia and liver failure. In cerebral ischemia, the damaged neurons accumulate abnormal intracellular fluid and ion-channel dysfunction leading to vascular injury leading to vascular damage (35). Cerebral ischemic tissue leads to a significant reduction in glucose supply, causing decreased intracellular ATP production. Prolonged hypoxic environment accelerates intracellular ATP depletion, leading to failure of sodium and calcium ion transport systems across cell membranes and excessive accumulation of intracellular sodium and calcium ions. Increased intracellular sodium causes abnormal entry of extracellular fluid into cells, resulting in cellular swelling (35). Abnormal accumulation of calcium ions prompts cells to initiate cytotoxic programs, triggering inflammatory responses by activating c-fos and c-jun genes and related cytokines, activating microglial cells to release free radicals and proteases, and damaging capillary membrane structures (36). After cytotoxic edema forms, the body accelerates sodium ion excretion from blood vessels to maintain sodium and water balance in extracellular fluid (37). The process of transferring sodium ions from intravascular to extracellular fluid causes intravascular fluid to seep into extracellular fluid, resulting in accumulation of extracellular fluid, known as ionic edema (31). Therefore, cytotoxic edema also leads to increased intracranial pressure.

## 3 Influencing factors and mechanisms of peritumoral edema

**Figure 2:**
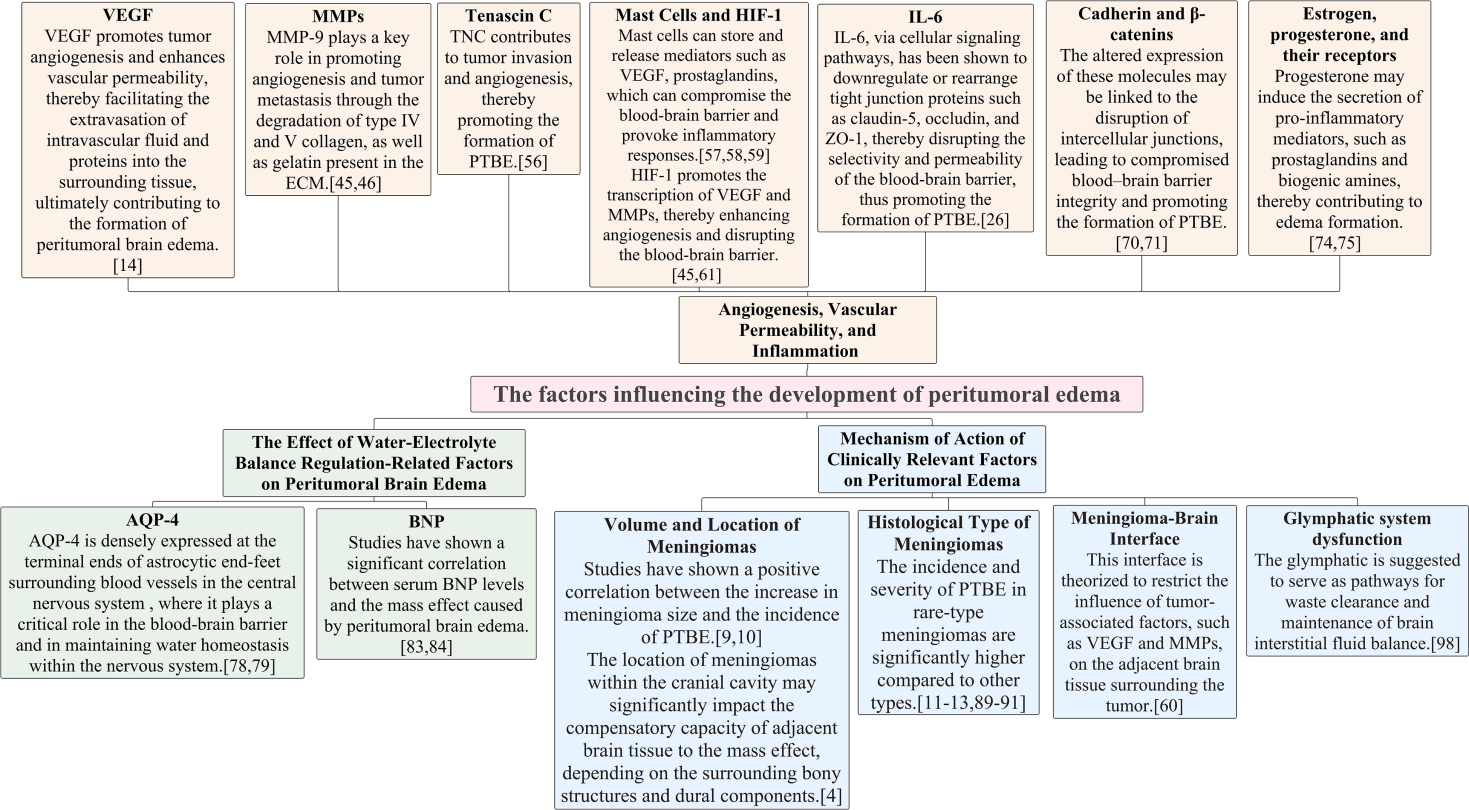
Overview of factors affecting PTBE in meningioma patients. VEGF, Vascular Endothelial Growth Factor; MMPs, Matrix Metalloproteinases; HIF-1, Hypoxia-Inducible Factor-1; IL-6, Interleukin-6; AQP-4, Aquaporin-4; BNP, Brain natriuretic peptide.

**Figure 3:**
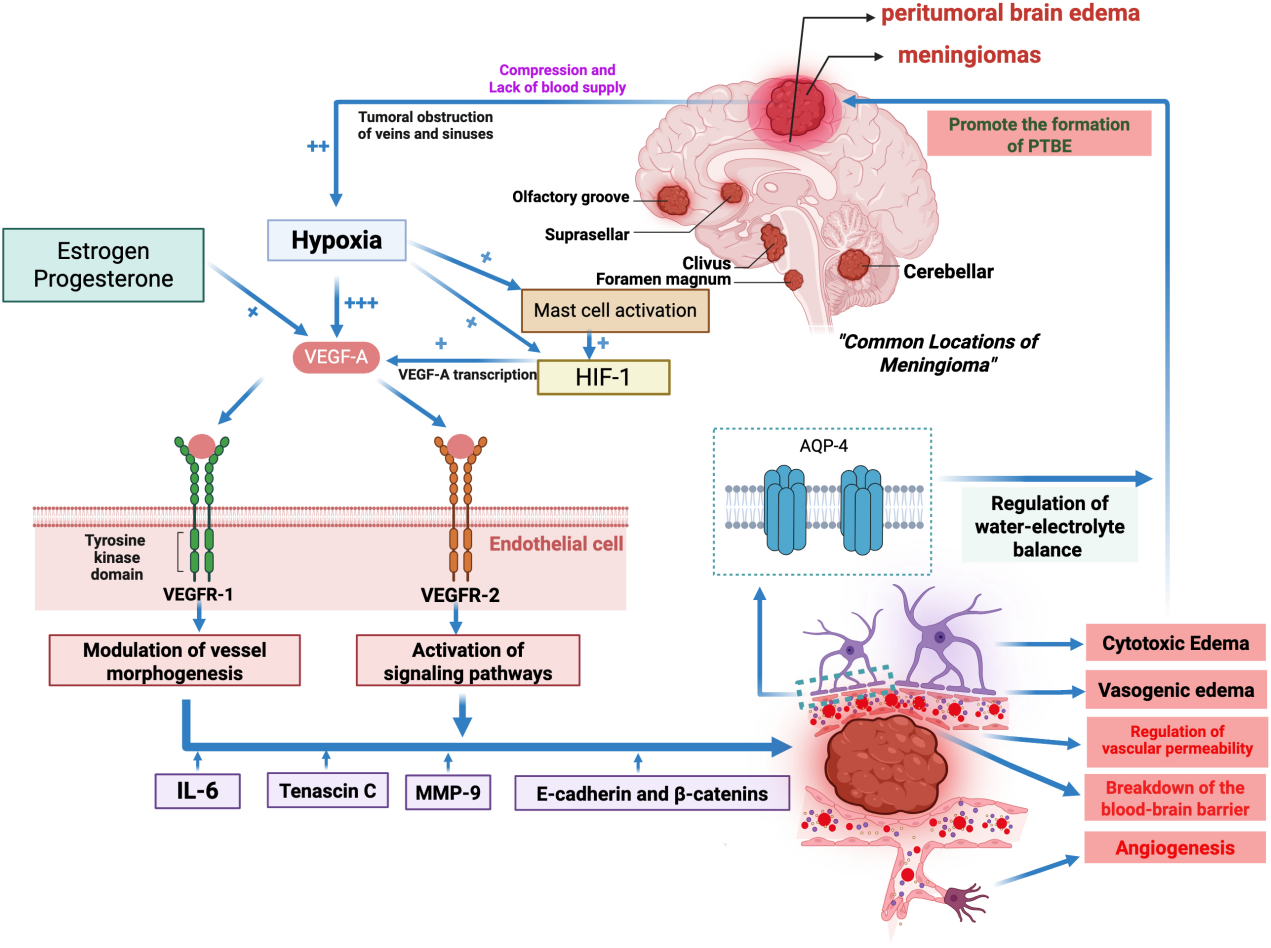
Overview of the interaction mechanisms among various influencing factors under hypoxic conditions. Meningiomas are frequently associated with PTBE, whose severity depends on tumor location, size, and histological subtype. The combined mass effect of the tumor and surrounding edema compresses adjacent brain tissue, blood vessels, and venous sinuses, leading to local hypoxia. Hypoxia increases VEGF-A expression through HIF-1–dependent signaling and is further modulated by mast cell activation as well as estrogen and progesterone. VEGF-A promotes angiogenesis and vascular permeability through VEGFR-1 and VEGFR-2, while other mediators such as IL-6, tenascin C, MMP-9, cadherins and β-catenin, and AQP-4 impair BBB integrity, extracellular matrix stability, and water–electrolyte balance, thereby contributing to PTBE formation.

In meningiomas, based on level 4 evidence, the influencing factors leading to peritumoral edema in patients are summarized in **Figure 2**. Under hypoxic conditions, the interaction mechanisms among various influencing factors are briefly summarized in **Figure 3**.

### 3.1 Mechanisms of angiogenesis, vascular permeability, and inflammation-related factors in the formation of PTBE

#### 3.1.1 Vascular endothelial growth factor

Vascular endothelial growth factor (VEGF) operates as a highly selective heparin-binding molecule that targets vascular endothelial cells, where it orchestrates both angiogenic processes and modulation of vascular permeability through receptor-mediated mechanisms (38). The family of VEGF genes includes several ligands, such as VEGF-A, VEGF-B, VEGF-C, VEGF-D, and placental growth factor. Among these, VEGF-A serves as the principal mediator of angiogenic processes (39). These ligands exert their biological effects by interacting with three different receptor tyrosine kinases, namely VEGFR-1, VEGFR-2, and VEGFR-3 (40–42). VEGFR-1 and VEGFR-2 are expressed on the surface of the vascular endothelium (40, 43), and VEGFR-3 has a preferential expression in the lymphatic endothelial network (44). VEGF binding to VEGFR-2 activates subsequently implemented downstream signaling pathways, especially through the PI3K/AKT pathway, which is strongly linked to tumor-associated angiogenesis and related alterations in vascular permeability (14). The mechanism by which VEGF binds to VEGFR-1 is unclear, but it appears to play a role in pathological angiogenesis of adult tumors and inflammation, and the regulation of vascular morphogenesis (14). Tumor development is often accompanied by dysregulation of VEGF expression, which affects the formation of PTBE. Schmid et al. (15) found that VEGF plays the main role in the neovascularization of meningiomas and that VEGF expression in soft meningeal blood supply is closely related to PTBE. Similarly, Nassehi et al. (16), in a study of 101 meningioma cases, found that 43 patients had PTBE, which correlated positively with VEGF expression. Another study (45) observed that varying degrees of VEGF expression in meningioma tissue exhibit a significant association with tumor grade and are detectable throughout all phases of peritumoral brain edema. Collectively, VEGF stimulates the growth of tumor-associated blood vessels and increases vascular permeability. This results in the leakage of fluid and proteins from blood vessels, thereby playing a part in the generation of brain edema around tumors.

#### 3.1.2 Matrix metalloproteinases

Matrix metalloproteinases (MMPs) are zinc-dependent enzymes mainly produced by tumor cells that degrade extracellular matrix and basement membrane components, aiding tissue remodeling. MMP-9 is the largest enzyme by molecular weight in the MMP family and specifically promotes angiogenesis and tumor metastasis by degrading type IV and V collagen and gelatin in the extracellular matrix (45, 46). In addition to structural effects, evidence indicates that MMP-9 contributes to PTBE by influencing the MAP kinase signaling pathway and activating glial cells, which increase local inflammation (21, 22). In a comprehensive analysis by Reszec et al. (45), MMP-9 was found in 65 of 93 meningioma samples, with higher levels in atypical, malignant tumors, and in patients with grade II or III PTBE. Consistent with these findings, Jung et al. (47) also confirmed that MMP-9 levels correlate with PTBE severity. The evidence indicates that MMPs are closely linked to PTBE and can be used as a predictive factor for the severity of PTBE.

#### 3.1.3 Tenascin C

Multiple different angiogenic factors exist in meningiomas, such as platelet-derived growth factor and VEGF (48–50). The interactions of these factors within the tumor’s extracellular matrix cannot be separated from other crucial factors. Tenascin C is a glycoprotein family widely present in the human extracellular matrix, which, although rarely expressed in normal adult tissues, participates in embryonic development, injury, inflammation, wound repair, and tumor angiogenesis (51, 52). Multiple studies have established that tenascin C expression is strongly associated with tumor angiogenesis (53, 54). Research has found that Tenascin C enhances the motility of vascular endothelial cells and stimulates endothelial cell proliferation by binding integrin receptors and activating the MAPK signaling pathway (53, 55). In a retrospective examination of 20 typical, 20 atypical, and 5 malignant meningioma cases, Kilic et al. (56) observed that Tenascin C expression within meningiomas correlated with both VEGF levels and the presence of PTBE. They speculated that Tenascin C participates in tumor invasion and tumor angiogenesis, promoting the occurrence of PTBE. However, the specific mechanism of Tenascin C in meningioma-related PTBE lacks subsequent research for confirmation.

#### 3.1.4 Mast cells and hypoxia-inducible factor-1

Meningiomas develop within a complex immune microenvironment that includes mast cells, macrophages, T and B lymphocytes (57). Studies have found that mast cells can store and secrete mediators such as VEGF, prostaglandins, and substance P (57–59), and the release of these mediators can disrupt the BBB and cause inflammatory responses (57, 59). Polyzoidis et al. (57) and colleagues reported mast cell infiltration in nearly 90% of high-grade meningiomas. Building on this, Reszec et al. (60) proposed a simplified classification dividing tumors into low-grade (WHO grade I) and high-grade (WHO grade II and III). The results showed that mast cells appeared in 31.8% of the low-grade group, with all cases also displaying significant PTBE. In contrast, 86% of the high-grade group contained mast cells, and all of these cases also showed PTBE. Moreover, there is a possible connection between mast cell activation and the regulation of HIF-1 overexpression in hypoxic conditions (60).

HIF-1 is a heterodimeric transcription factor composed of HIF-1α and HIF-1β. Under normoxia, HIF-1α is hydroxylated and degraded, but in hypoxia, it stabilizes, moves to the nucleus, and binds HIF-1β to activate genes (like VEGF, MMPs), promoting angiogenesis, glycolysis, and cell survival (61). In a hypoxic microenvironment, HIF-1 attaches to sites in the VEGF enhancer, leading to increased VEGF transcription via the PI3K/Akt pathway (62). It also stabilizes VEGF and its receptors, which promotes angiogenesis (63). Related studies indicate that HIF-1 is detected in 55.7% of low-grade and 84% of high-grade meningiomas (64). MMP is another important target gene of HIF-1α, and HIF-1 can directly or indirectly induce the expression or activation of MMPs, leading to basement membrane degradation and impaired function of tight junction proteins (45). Therefore, hypoxia can promote mast cell activation and HIF-1 overexpression, resulting in the destruction of the BBB, thereby triggering PTBE.

#### 3.1.5 Interleukine-6

Interleukin-6 (IL-6) is a pro-inflammatory factor produced by macrophages during tissue injury or inflammation (65). It is playing a pivotal role in helping mediate inflammation, cell differentiation, and immune responses in many cell types, including tumor cells, endothelial cells, and astrocytes (66). The role of IL-6 in the CNS continues to be debated. Trans-signaling involves the classic membrane-bound IL-6 receptor or the soluble IL-6 receptor (sIL-6R). It activates downstream pathways (like JAK/STAT, Ras/MAPK, and PI3K/AKT), which regulate cell survival, proliferation, the inflammation process, and stimulate angiogenesis (23). However, in a study by Todo et al. (24), exogenous low-dose IL-6 was shown to inhibit thymidine incorporation in meningioma cells, suggesting that IL-6 secreted by meningiomas might act as a local growth inhibitory signal, rather than a growth promoter. In a case series reported by Park et al. (25), on investigating the PTBE volume in 36 patients with benign meningioma, it was noted that 12 out of 16 patients with PTBE were positive for IL-6, whereas only 6 out of 20 patients without edema were positive. Notably, the IL-6 level in the edema group was 7.72 times higher than in controls, and PTBE severity was significantly linked to IL-6 expression. Recent research can provide a mechanistic explanation for the above findings: IL-6 leads to decreased expression or rearrangement of tight junction proteins through cellular signaling pathways, thereby disrupting the selectivity and permeability of the BBB and promoting the formation of PTBE (26).

#### 3.1.6 Cadherins and β-catenin

Cadherins are a family of glycoprotein receptors that are either transmembrane or attached to the membrane. They enable calcium-dependent cell adhesion and are crucial for the synchronized development of different tissues and organs (67). Among the molecules closely associated with cadherin-mediated adhesion, β-catenin functions as a multifunctional signaling protein. In epithelial tissues, E-cadherins interact with β-catenin molecules to form adhesive structures that are involved in the maintenance of junction stability, as well as in the regulation of signaling pathways (67). It is through this regulatory function that it indirectly influences the proliferation of tumor cells and the transmission of cellular signals (67–69). Accumulating research suggests that cadherins are probably a pivotal and determinative segment in the invasive behavior of meningiomas and the degree of associated PTBE. As one illustration, Zhou et al. (70) noted that higher tumor grade in meningiomas is associated with reduced E-catenin and β-catenin, which is almost absent in malignant types. This describes a decrease in cell adhesion with tumor progression. Another investigation by Rutkowski et al. (71) revealed that in 154 intracranial meningioma instances, N-cadherin was remarkably upregulated in high-grade tumors, with the accumulation of β-catenin happening in the cell nuclei. Importantly, both the molecular alterations correlated strongly with postoperatively evaluated levels of PTBE, and this correlation was particularly pronounced in tumors of more aggressive grades. Collectively, these findings indicate a consistent association between disruptions in cadherin and β-catenin signaling pathways and the progression of PTBE severity. It has therefore been proposed that dysregulation of cadherins and β-catenin contributes to the disruption of intercellular junctions, weakening BBB integrity, and promoting the formation of PTBE.

#### 3.1.7 Estrogen, progesterone, and their receptors

Sex hormones, particularly estrogen and progesterone, together with their corresponding receptors, have long been implicated in the initiation and progression of meningiomas, as well as in shaping the tumor microenvironment. Evidence shows progesterone receptors are common in meningiomas, while estrogen receptors are rare, usually below 10% or absent (72). Some studies have indicated that progesterone receptor expression levels are higher in benign meningiomas than in atypical or anaplastic meningiomas. They speculate that progesterone receptor expression levels are closely related to the type and prognosis of meningiomas, with higher progesterone receptor expression indicating better prognosis, while meningiomas with lower progesterone receptor expression or the presence of estrogen receptor expression may suggest poor prognosis (73). In early studies on brain edema and sex hormone positivity, Maiuri et al. (74) found that progesterone may induce the secretion of pro-inflammatory markers, thereby causing brain edema. A recent study found that among 22 meningioma cases, 19 cases showed progesterone positivity and all exhibited significant PTBE (75). Although the precise mechanisms linking sex hormones and their receptors to PTBE in meningioma remain unclear, current evidence suggests that sex hormones may represent a potential therapeutic target. Further investigation into their mechanistic roles is therefore warranted.

### 3.2 The effect of water-electrolyte balance regulation-related factors on peritumoral brain edema

#### 3.2.1 Aquaporins

Aquaporins (AQPs) are a group of transmembrane channels found on the plasma membranes of major human organs and tissues (76). AQP-4 is currently the most water-permeable aquaporin known and serves as the main water channel protein in the mammalian CNS (77). Its localization is very polarized, and there is dense expression at astrocytic end-feet covering cerebral microvessels, placing AQP-4 at the crucial boundary between the brain tissue and blood vessels (77, 78). Through this distribution, AQP-4 is important in maintaining BBB function and water balance within the CNS (78). The specific mechanisms by which AQP-4 is involved in PTBE development in meningiomas are still incompletely understood. Accumulating evidence suggests there is a strong correlative association between high AQP-4 and the presence and severity of PTBE. Elevated AQP-4 in meningioma tissues is also linked to VEGF, suggesting involvement in the formation of edema in a pro-angiogenic and permeability-enhancing environment (17–19). Experimental studies further indicate that the role of AQP-4 may differ based on the type of cerebral edema. In mouse models of cytotoxic edema, using inhibitors to block AQP-4 activity has been demonstrated to reduce both the formation and severity of brain swelling (79, 80). In contrast, loss of AQP-4 activity in vasogenic edema appears to exacerbate tissue swelling, a phenomenon attributed to impaired clearance of excess interstitial fluid from the brain tissue (76, 81, 82). Furthermore, other aquaporin subtypes may also participate in PTBE pathogenesis. Lambertz et al. (20) found that AQP-5 is expressed in meningiomas and is correlated with the occurrence and severity of PTBE, providing data to support its involvement in the maintenance of brain water balance.

#### 3.2.2 Brain natriuretic peptide

Brain natriuretic peptide (BNP) is primarily produced by the myocardial cells of the dilated ventricles. After binding to its receptors, BNP exerts regulatory effects on water–electrolyte homeostasis by suppressing sympathetic nervous system activity and modulating the renin–angiotensin–aldosterone axis. In clinical practice, BNP is commonly regarded as a biomarker for heart failure and also as an indicator of hypoxia. In the CNS, BNP receptors are widely distributed. Clinical studies have reported a strong link between circulating BNP levels and the mass effect induced by PTBE (83, 84). Ruggieri et al. (84) demonstrated that serum BNP levels in patients with brain tumors are positively correlated with PTBE volume. Nevertheless, the precise mechanism underlying this correlation remains unknown, and it is uncertain whether BNP is merely a byproduct of PTBE or if it actively contributes to its pathogenesis. Still, serum BNP levels in brain tumors can indicate the effectiveness of anti-edema drug therapy (84).

### 3.3 Mechanism of action of clinically relevant factors on peritumoral edema

#### 3.3.1 Volume and location of meningiomas

Existing studies have shown that meningioma-related PTBE is strongly associated with the volume and location of meningiomas. Across multiple clinical studies, a consistent positive relationship has been observed between increasing meningioma volume and the likelihood of PTBE development (9, 10). In a study by Shin et al. (9), a retrospective study of 205 patients with convexity or parasagittal meningiomas found that a tumor size of 13.95 cm³ (or a tumor diameter of 2.99 centimeters) as a cutoff value could predict the incidence of PTBE, with a sensitivity of approximately 76.1% and a specificity as high as 92.5%. In an earlier study by Tamiya et al. (85), in addition to observing a correlation between tumor size and PTBE volume, it was found that convex meningiomas and middle fossa meningiomas had the highest average tumor edema index (PTBE volume/tumor volume). Moreover, the most severe PTBE occurred in convex meningiomas infiltrating the middle fossa. Recently, Liyanage et al. (10) further confirmed that both the mass effect and the probability of PTBE increased with the tumor’s maximum diameter. Furthermore, significant PTBE was observed when meningiomas involved the supratentorial and infratentorial spaces. A plausible explanation for these observations lies in the heterogeneity of intracranial anatomy. The ability of surrounding brain tissue to accommodate tumor-related mass effect varies according to local bony confines and dural structures, which differ markedly across cranial regions. When this compensatory capacity is limited, even moderate tumor enlargement may precipitate disproportionate edema formation (4).

#### 3.3.2 Histological type of meningiomas

Currently, meningiomas are divided into 15 subtypes. Of these, WHO grade I comprises 9 subtypes, WHO grade II consists of 3 subtypes, and WHO grade III includes 3 subtypes (3) (**Table 1**). In early studies on the correlation between meningioma histological types and PTBE, Osawa et al. (86) classified nine subtypes of WHO grade I meningiomas into common types (meningothelial, transitional, and fibrous meningiomas) and rare types (psammomatous, angiomatous, microcystic, secretory, lymphoplasmacyte-rich, and metaplastic meningiomas). In a retrospective study of 110 patients with meningiomas, they found that rare-type meningiomas were associated with extensive PTBE, with PTBE volumes ranging from 10 to 100 times those of other common-type meningiomas. In subsequent studies, the incidence and severity of PTBE in angiomatous and secretory meningiomas were significantly higher than in other meningioma types (11–13). Although the mechanisms underlying significant PTBE in specific subtypes of meningiomas are not yet fully understood, multiple studies have proposed potential mechanisms.

**Table 1:** Histological subtypes of meningioma, histological features, and edema frequency in each subtype.

WHO grading of meningiomas	Subtypes	Histopathological features	Edema frequency
HWO grade I	Meningothelial meningioma	Sheets or lobules of meningothelial cells, Relatively rich capillary network, Abundant collagenous stroma	Moderate
	Fibrous meningioma	Well-preserved arachnoid plane, Relatively sparse vascularity	Low
	Transitional meningioma	Combined meningothelial and fibrous patterns	Moderate
	Psammomatous meningioma	Dominated by numerous psammoma bodies, Relatively low microvascular density	Low
	Angiomatous meningioma	Numerous, dilated thin-walled blood vessels	High
	Microcystic meningioma	Prominent microcystic spaces between tumor cells, High vascular permeability	High
	Secretory meningioma	PAS-positive pseudopsammoma bodies, Strong induction of vascular permeability	High
	Lymphoplasmacyte-rich meningioma	Mature lymphocytes, Cytokine-rich milieu	High
	Metaplastic meningioma	Heterogeneous composition, Relatively low vascular density	Low
HWO grade II	Chordoid meningioma	Increased cellularity, Elevated mitotic activity, Necrosis and brain invasion, Destruction of the arachnoid barrier, Severe blood–brain barrier disruption	High
	Clear cell meningioma		
	Atypical meningioma		
HWO grade III	Papillary meningiomas		
	Rhabdoid meningiomas		
	Anaplastic meningioma		

For example, angiomatous meningioma is a highly vascularized benign meningioma, and high vascularization may be related to VEGF expression in meningiomas (14, 87). Angiomatous meningioma may produce abnormally extensive PTBE due to the combined effects of its high vascularization and VEGF. Secretory meningiomas contain periodic acid-Schiff positive pseudopsammoma bodies, and this subtype has a higher number of mast cells compared with other meningioma subtypes (88). VEGF, prostaglandins, tumor necrosis factor, and substance P, which are stored and secreted by mast cells, aggravate the degree of PTBE by disrupting the BBB (59). The main characteristics of microcystic meningioma include vacuolization, myxoid degeneration, and microcyst formation, with suspected excessive secretory activity of tumor cells (89). Microcystic meningioma also exhibits high vascularity, and VEGF immunoreactivity in tumor endothelial cells is higher than in other common meningiomas (90). The high degree of vascularization and strong expression of VEGF may be the cause of significant PTBE associated with microcystic meningioma. Lymphoplasmacyte-rich meningioma is characterized by extensive infiltration of inflammatory cells and varying proportions of meningioma cells, with the infiltrated lymphoplasmacytes potentially obscuring the meningothelial cell composition (3). A large infiltration of non-neoplastic lymphocytes and plasma cells can trigger an inflammatory response, which may be key in the development of PTBE linked to lymphoplasmacyte-rich meningioma (91).

#### 3.3.3 Meningioma-brain interface

Meningiomas typically possess a distinct boundary composed of the pia–arachnoid and tumor matrix, known as the meningioma–brain interface. Theoretically, this interface limits the effects of tumor-related factors such as VEGF and MMPs on adjacent peritumoral brain tissue (60). Therefore, identifying factors that can disrupt this interface is crucial for understanding the pathogenesis of PTBE. From a macroscopic perspective, the integrity of the meningioma-brain interface directly affects the formation of PTBE. In a study by Nakasu et al. (92) involving 50 meningioma surgical cases, it was found that the extent of arachnoid rupture correlated with peritumoral edema. At the microscopic level, Huang et al. (93) utilized single-cell RNA sequencing to analyze cell types with distinct functions and molecular characteristics in meningioma-associated tissues. They identified specific tumor cell subpopulations within the meningioma-brain interface microenvironment that promote tumor angiogenesis. Tumor angiogenesis has been closely linked to the development of PTBE (94).

#### 3.3.4 Glymphatic system dysfunction

For decades, the lack of classical lymphatic vessels within the brain parenchyma led to the prevailing view that protein and metabolic waste clearance depended primarily on intracellular and extracellular degradation pathways, including autophagy and ubiquitin-mediated proteolysis (95, 96). Under this framework, Only a few proteins, such as amyloid-β, can cross the BBB and be cleared by specific transporters (97). As research has advanced, some scholars suggest that features of the glymphatic and meningeal lymphatic systems exist in humans, with the glymphatic system potentially serving as a waste removal pathway and maintaining fluid balance in the brain parenchyma (97). In this context, Toh et al. (98) employed the analysis along the perivascular space (ALPS) index as an imaging surrogate of glymphatic function in a cohort of 80 patients with meningiomas. Their analysis showed a negative correlation between PTBE volume and the ALPS index, indicating that reduced glymphatic activity was associated with more extensive peritumoral edema. Such findings are consistent with the clinical observation that impaired fluid clearance often accompanies edema progression in intracranial tumors.

## 4 Surgical treatment of meningiomas and resolution of postoperative PTBE

Maximum safe surgical resection continues to be the preferred approach for meningioma and is essential for eliminating PTBE. The therapeutic mechanism works through: complete tumor resection that directly relieves mechanical compression of adjacent brain tissue by the tumor, and more importantly, blocks the secretion source of vascular permeability factors such as VEGF, thereby suppressing edema formation from its etiopathological basis (8, 99).

The regression of postoperative PTBE is a dynamic process. An early classic study calculated that the average regression rate of edema fluid through white matter surrounding a 1 cm³ tumor was 0.0493 ml/day, suggesting that 50% of edematous white matter may regress within 4 days after meningioma resection, and 90% may regress within 14 days (100). Not all PTBE resolves quickly after surgical resection, and its specific mechanisms remain unclear. Precise microsurgical technique is the key determinant of postoperative cerebral edema outcome. Intraoperatively, excessive traction, disruption of the arachnoid interface, or injury to the dural sinuses and draining veins should be avoided to prevent exacerbation of iatrogenic cerebral edema (101, 102). The malignancy grade of meningiomas is also a factor affecting postoperative edema resolution. Studies show that non-benign tumors, due to their tumor invasiveness and higher baseline edema, may lead to aggravation or difficulty in resolution of postoperative edema (103–105). Furthermore, postoperative cerebral edema outcomes may vary among different subtypes of WHO Grade I meningiomas. For example, angiomatous meningiomas are characterized by high vascularity and elevated VEGF levels (14, 87). Abundant vasculature and increased vascular permeability can aggravate surgical trauma, leading to postoperative edema (106). Secretory meningiomas are rich in mast cells, and surgical resection may trigger the release of vasoactive substances by activating mast cells, resulting in postoperative cerebral edema aggravation (88, 106, 107). In a study of 44 secretory meningiomas by Regelsberger et al. (103), severe postoperative cerebral edema was observed in 15 patients, with some cases showing progressive worsening. Notably, rare subtypes of WHO grade I meningiomas are often accompanied by significant preoperative peritumoral edema, and extensive peritumoral edema can increase surgical trauma and exacerbate postoperative cerebral edema (104, 106). In a recent study, Hu et al. (106) found that preoperative peritumoral edema is an independent risk factor for postoperative progressive cerebral edema and hemorrhage. Furthermore, research by Laajava et al. also found that persistent PTBE following complete resection of intracranial meningiomas is a common phenomenon, but typically shows significant regression (108). Currently, the nature and exact etiology of brain parenchymal changes associated with persistent postoperative PTBE remain unclear, but glial proliferation may be a potential etiological explanation that could provide direction for future research (108).

## 5 Radiotherapy for meningioma and aggravated cerebral edema after radiotherapy

### 5.1 Radiotherapy for meningioma

Meningiomas often develop near vital neural and blood vessels, which can restrict the possibility of complete surgical removal. In such scenarios, radiotherapy has become an increasingly important part of clinical treatment. At present, it is broadly regarded as a first-line treatment for small- to medium-sized meningiomas under 35 mm that show no symptoms, as well as for skull base lesions where surgery is limited by the need to protect critical structures like the optic chiasm and nerves (109, 110). Beyond its role as definitive therapy, radiotherapy is also commonly employed in the adjuvant setting to enhance local tumor control following subtotal resection or in cases of malignant meningiomas (111). Although patients with benign meningiomas generally have a favorable outlook following radiotherapy alone, the efficacy of radiotherapy in treating atypical and malignant meningioma patients remains controversial (112, 113). From a safety perspective, radiotherapy is not without risk. Clinical observations indicate that the majority of treatment-related toxicities involve cranial nerve dysfunction as well as the exacerbation or *de novo* development of cerebral edema following irradiation (114, 115). Although the precise molecular mechanisms underlying radiation-induced injury remain incompletely defined, accumulating evidence implicates radiation necrosis and complex inflammatory cascades characterized by elevated expression of VEGF and HIF-1 in the pathogenesis of post-radiotherapy cerebral edema (111).

### 5.2 Aggravated cerebral edema after radiotherapy

The duration of cerebral edema after radiotherapy ranges from 1 to 13 months, and symptoms in most patients can be relieved after standardized stepwise treatment (111). Corticosteroids remain the first-line pharmacological therapy for post-radiotherapy cerebral edema, with dexamethasone being the most commonly used medication, which alleviates vasogenic cerebral edema by inhibiting VEGF expression, stabilizing vascular endothelial cells, and reducing vascular permeability (111, 116, 117). Bevacizumab is a monoclonal antibody against VEGF that can effectively reduce PTBE and post-radiotherapy cerebral edema. For patients who are resistant to corticosteroid treatment or unable to tolerate corticosteroid side effects, bevacizumab can be used as a second-line treatment option (111, 118). Furthermore, magnetic resonance-guided laser interstitial thermal therapy (LITT) is a minimally invasive treatment technique developed in recent years, suitable for patients in whom drug therapy has failed and who are not candidates for surgery (119). By stereotactically placing a laser fiber and performing precise thermal ablation of the lesion under real-time magnetic resonance monitoring, it can effectively control post-radiotherapy cerebral edema. However, its long-term efficacy and safety require further clinical investigation and validation.

## 6 Common treatment drugs and therapeutic prospects for peritumoral edema

**Figure 4:**
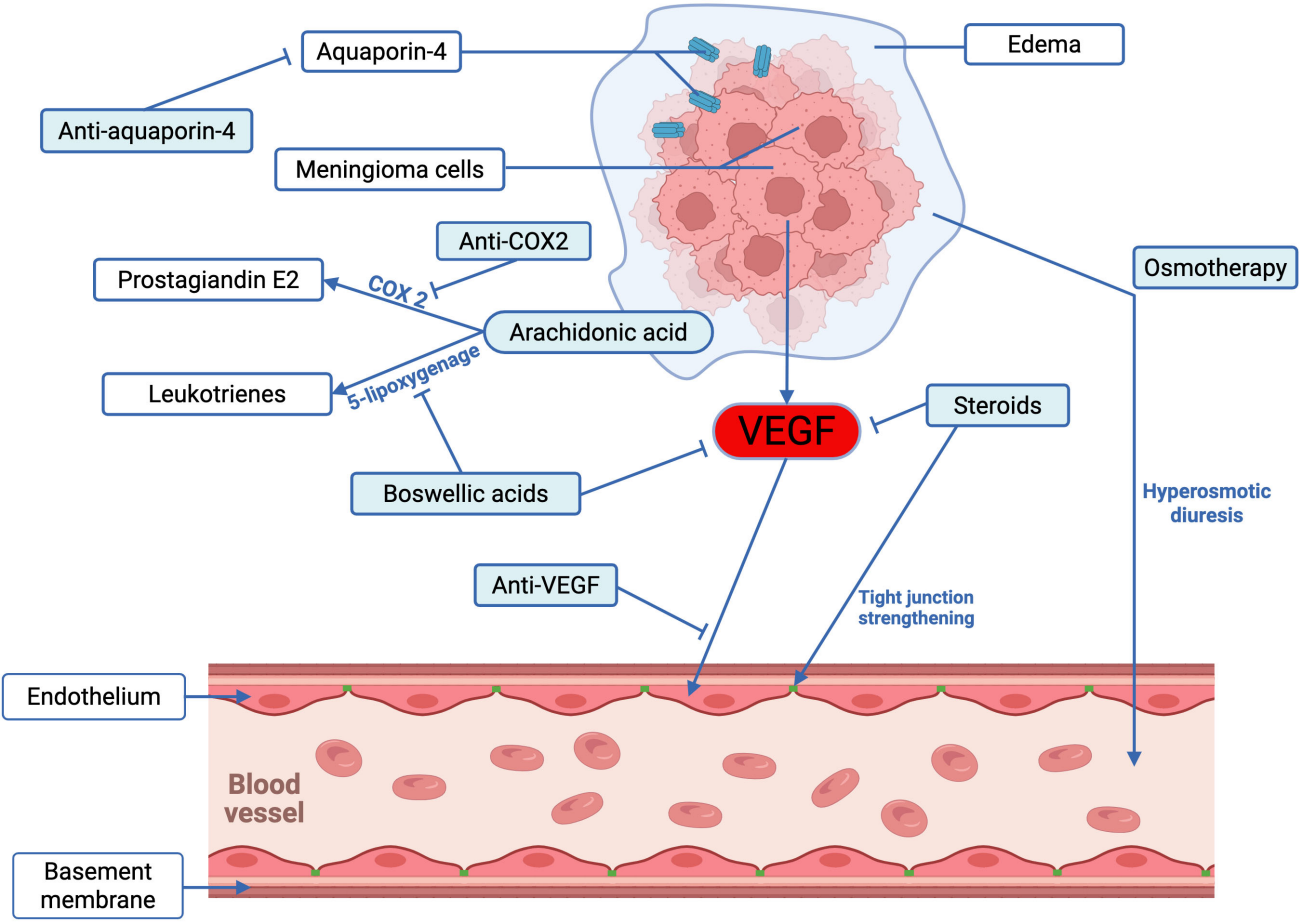
The mechanisms of action in drugs used to treat meningioma-related edema. Several drugs for the treatment of peritumoral brain edema have been clinically applied, as outlined below: (1) Osmotherapy with mannitol or hypertonic saline rapidly reduces intracranial pressure by increasing intravascular osmotic pressure, although the effect is short-lived. (2) Corticosteroids such as dexamethasone and prednisone remain standard treatments, as they reduce vascular permeability by inhibiting VEGF expression and restoring tight junction proteins; however, long-term use is limited by significant side effects. (3) Anti-VEGF therapies effectively decrease capillary permeability and tumor angiogenesis but are costly and associated with notable adverse effects. (4) Targeting aquaporin-4 (AQP-4) is another approach, as its overexpression is linked to edema formation; Goreisan, a traditional herbal medicine, may alleviate edema by downregulating AQP-4. (5) COX-2 inhibitors can reduce prostaglandin E2–mediated vasodilation and angiogenesis, though their cardiovascular risks restrict clinical use. (6) Boswellic Acid, particularly its active component AKBA, exhibits anti-inflammatory properties and VEGF inhibition, contributing to edema reduction.

The medications frequently employed in the clinical management of PTBE are summarized in **Table 2**, and a simplified schematic representation of their mechanisms of action in meningioma-associated edema is shown in **Figure 4**.

**Table 2:** List of drugs used to treat PTBE surrounding meningiomas.

Drug types	Typical drugs	Mechanism	Efficacy	Adverse event
Osmotherapy	Mannitol	Hyperosmotic diuresis	Rapid effect	A temporary effect only, Electrolyte imbalance
Steroids	Dexamethasone	Strengthened Tight junction and suppression of increased vascular permeability	Standard treatment	Infection, Immunosuppression, Multiple side effects
Anti-VEGF	Bevacizumab	Suppression of VEGF, Inhibition of vascular permeability and tumor angiogenesis	Strong effect	Hemorrhage, embolism, high drug cost
Anti-aquaporin 4	Goreisan	Control water accumulation	Unknown	Unknown
Anti-COX-2	Celecoxib	Inhibition of prostaglandin synthesis → Reduction of vascular permeability	Unknown	Cardiovascular disease
Boswellic acids	5-Loxin	Interference with the VEGF pathway and leukotriene formation	Unknown	Gastrointestinal side effects

COX-2: cyclooxygenase-2.

### 6.1 Osmotherapy

In clinical practice, mannitol and hypertonic saline are the main osmotic therapy agents. They are especially used in persons who are at imminent risk of raised intracranial pressure or brain herniation (27, 120). They increase intravascular osmotic pressure, which draws water from the oedematous brain tissue into the bloodstream to reduce intracranial hypertension. Intravenous mannitol and hypertonic saline can help to rapidly reduce the pressure in the brain, often in minutes to half an hour. However, the therapeutic benefit is temporarily limited by definition as pressure-lowering effects are only usually lasting a few hours (27). Prolonged use of these agents is linked to negative reactions, such as electrolyte disturbances and acute renal injury (28). As such, osmotherapy is generally considered a short-term, emergency treatment option in clinical settings.

### 6.2 Corticosteroids

Corticosteroids are generally regarded as the usual treatment for PTBE in the perioperative period (29), with the effects for moderate to severe PTBE being particularly noticeable. However, there is great variability in the perioperative corticosteroid regimens of neurosurgeons for meningioma patients. The most frequent regimen expressed is that of dexamethasone, at a daily dose of 16 mg in four divided doses (121). Prednisone and dexamethasone, by virtue of their power and mineralocorticoid effect, are often used as example corticosteroids for the treatment of tumor-associated edema with significant efficacy in the treatment of vasogenic edema (4, 122). The therapeutic effects of corticosteroids are thought to arise through the inhibition of VEGF expression, a reduction in vascular permeability to permeability factors, and the repair or enhancement of tight junction protein expression (such as claudins, occludins, and cadherins), thereby reducing gaps and permeability at the meningioma-brain interface (29, 123). Additionally, corticosteroids, when combined with blood pressure and fluid management measures, can rapidly lower intracranial pressure when PTBE results in significant pressure increases or severe neurological deficits (122, 124). However, clinical use requires high-dose corticosteroids to effectively reduce peritumoral edema, and long-term use may lead to serious complications, including immunosuppression, increased infection risk, avascular necrosis of the femoral head, Cushing’s syndrome, and osteoporosis (29).

### 6.3 Anti-VEGF agents

VEGF is widely recognized as a central mediator in the development of PTBE, exerting its effects primarily by increasing vascular permeability and driving tumor-associated angiogenesis (8). Unlike conventional inflammatory mediators, VEGF exerts an exceptionally potent permeability-enhancing effect, reported to be nearly 1,000 times greater than that of histamine, by activating tyrosine kinase receptors on capillary endothelial cells (123).

Bevacizumab (a monoclonal antibody targeting VEGF) and sunitinib (a multi-target tyrosine kinase inhibitor acting on VEGF receptors) have both been reported to reduce tumor-associated PTBE (118, 125). These drugs can reduce capillary permeability and inhibit tumor angiogenesis, showing significant effects in treating PTBE. However, their clinical application must take into account the high treatment costs and severe side effects, such as hemorrhage, thrombosis, and hepatotoxicity (118, 125). At present, bevacizumab is approved by the US Food and Drug Administration for the treatment of recurrent malignant brain tumors (126, 127). However, proof for the application of anti-VEGF agents in meningioma-associated PTBE in a wide proportion is still lacking. There is an imminent need for prospective randomized control trials to assess the genuine efficacy and risk-benefit ratio of anti-VEGF drugs for both the holistic meningioma prognosis and control of PTBE.

### 6.4 Prospects for treatment of peritumoral edema

Considering the side effects, as well as the limited efficacy associated with permeation therapy and steroid treatment, exploring and developing new drugs and new therapies for PTBE is imperative. Among the many different treatment approaches currently under investigation, the following are some of the potentially valuable directions.

#### 6.4.1 Anti-AQP-4 agents

Currently, the specific mechanism of aquaporin formation with PTBE is not understood in full, and further research is needed in order to elucidate its role in PTBE formation (17–19, 77, 78). Among those channels, AQP-4 has gained special interest due to its prominent expression in astrocytic endfeet and its close relation to fluid homeostasis at the blood-brain interface. In this regard, Goreisan, a traditional herbal preparation widely used in Japan and other East Asian countries (known as Wu Ling San in China and Oreongsan in Korea), has been suggested as a possible cerebral edema modulator via its downregulatory effect on AQP-4 expression (128–130). Studies have shown that Goreisan helps to reduce brain edema in stroke (80). Parallel findings in animal models strengthen this view, since several studies have shown measurable improvements in brain edema following Goreisan administration (131, 132). These results hint that Goreisan may be helpful in treating PTBE, but more research would be needed to determine the therapeutic potential of Goreisan to be used as a treatment for PTBE.

#### 6.4.2 COX-2 inhibitors

COX-2 is expressed in meningiomas and can increase the production of prostaglandin E_2_ (133, 134). Prostaglandin E_2_ has vasodilatory effects, is associated with tumor angiogenesis, and may promote the formation of cerebral edema (133). COX-2 has garnered attention not only for its role in tumor angiogenesis but also for its association with tumor progression, metastasis, and the development of drug resistance (134). Despite their potential value, COX-2 inhibitors have been rarely studied in the research of meningioma-related peritumoral brain edema in the past period (135–137). Selective COX-2 inhibition has been linked to severe side effects, especially myocardial infarction and other cardiovascular issues, which have greatly limited their use in therapy (138). The underlying mechanisms responsible for these cardiovascular risks remain incompletely understood, which has led to the withdrawal of several COX-2 inhibitors from clinical practice (138, 139). Therefore, conducting safety assessments is essential for developing COX-2 inhibitors as a possible treatment for PTBE.

#### 6.4.3 Boswellic acids

Boswellic acid, a phytotherapeutic compound from frankincense resin, has gained growing interest mainly because of its key active component, acetyl-11-keto-β-boswellic acid (AKBA) (140). AKBA shows anti-inflammatory effects and suppresses VEGF, helping reduce brain edema (141, 142). AKBA is a powerful inhibitor for HIF-1α, resulting in inhibition of the downstream expression of VEGF and a reduction in the formation of brain edema. In the meantime, AKBA suppresses 5-lipoxygenase, which is important in the synthesis of leukotrienes from arachidonic acid, and greatly influences the permeability of the vascular wall (141). Clinical observations also help to strengthen these experimental insights. In a randomized study performed by Kirste et al. (143), 44 patients with malignant brain tumors received radiotherapy plus boswellic acid or placebo therapy. After checking the brain edema through MRI, it was noticed that 60% of the patients treated with boswellic acid showed a percentage greater than 75% in the reduction of the edema area, compared to 26% in the placebo group, which achieved a reduction in the area of edema of similar magnitude to the group of patients treated with boswellic acid. A recent study on 20 patients with glioblastoma treated with boswellic acid in combination with radiotherapy found that the administration of boswellic acid greatly reduced brain edema due to chemoradiotherapy (144). While the direct evidence in meningioma patients is currently lacking, the overlap in the pathways of the vascular permeability and the inflammatory signaling mediated by the molecule VEGF seems to make a therapeutic role probable. Therefore, it is thought that the boswellic acid may have some potential therapeutic benefits in treating meningioma-related PTBE.

## 7 Future perspectives

Osmometry, corticosteroid treatment, and anti-VEGF drugs have different side effects that can negatively affect the quality of life of patients. Anti-VEGF drugs (like bevacizumab) are expensive, and for treatment require an implant of a vein access device (123), affecting the quality of care for the patient. Therefore, it is imperative to develop effective PTBE treatments with fewer side effects and lower costs. Taking into consideration the above factors, the anti-AQP-4 drugs and boswellic acid were administered orally and are relatively inexpensive, so the combination therapy of anti-AQP-4 drugs and boswellic acid for the therapy of PTBE appears to have significant potential value (132, 140). Currently, there are some reports on the efficacy of these two treatments, but there is a dearth of evidence from long-term treatment outcomes and clinical trials for PTBE on a large scale. Larger-scale studies are required to confirm whether treatment with these two drugs has the potential to save costs and have superior efficacy as compared to steroid or anti-VEGF treatments. Additionally, a better understanding of the molecular mechanisms underlying AQP-4 and AKBA functional activity may help to optimize the therapeutic potential.

## 8 Conclusions

The incidence of PTBE associated with meningioma is still a global problem with both clinical and ethical urgency of concern, significantly affecting the clinical symptoms and long-term outcome of the patients. The occurrence of PTBE is closely linked to the permeability of the BBB and tumor angiogenesis as a result of an interaction between several factors. Osmotherapy and steroids are the primary treatments for PTBE, but there are side effects and a lack of effectiveness that cannot be ignored. Several studies show the potential and effectiveness of anti-VEGF drugs in reducing PTBE, but their high cost and severe side effects (bleeding and embolism) are major obstacles to widespread clinical use. Therefore, exploring and developing new drugs and new therapies for PTBE are of utmost urgency. New therapeutic strategies using anti-AQP-4 drugs and boswellic acid show promising prospects. Future deeper understanding of the molecular mechanisms of AQP-4 and AKBA functional activities may help fully realize their therapeutic potential, ultimately improving patient prognosis and treatment outcomes.
